# Profiles of emerging adults’ resilience facing the negative impact of COVID-19 across six countries

**DOI:** 10.1007/s12144-022-03658-y

**Published:** 2022-10-10

**Authors:** Sorgente Angela, Gabriela Fonseca, Žan Lep, Lijun Li, Joyce Serido, Rimantas Vosylis, Carla Crespo, Ana Paula Relvas, Maja Zupančič, Margherita Lanz

**Affiliations:** 1 Department of Psychology, Università Cattolica del Sacro Cuore, Largo Gemelli, 1 Milan (IT), 20123 Milan, Italy; 2 Faculty of Psychology and Education Sciences, Centre for Social Studies, University of Coimbra, Coimbra, Portugal; 3 Faculty of Arts, University of Ljubljana, Ljubljana, Slovenia; 4 Department of Family Social Science, University of Minnesota – Twin Cities, Minnesota, US; 5 Institute of Psychology, Mykolas Romeris University, Vilnius, Lithuania; 6 Faculty of Psychology, CICPSI, University of Lisbon, Lisbon, Portugal

**Keywords:** Resilience, ego-resiliency, Emerging adults, COVID-19, Latent profile analysis

## Abstract

Although emerging adults (i.e., individuals aged 18–29 years old) may be at a lesser risk of COVID-19 severe illness and mortality, studies have found that the negative impact of COVID-19 on mental health and well-being is higher among emerging adults when compared to other age groups. The current study aimed to identify profile(s) based on resilience resources, which could help emerging adults in managing the disruptions to their lives following the pandemic. A cross-national sample of 1,768 emerging adults from China, Italy, Lithuania, Portugal, Slovenia, and the US was utilized to identify profiles based on different resilience dimensions (ego-resiliency, positivity, religiosity, socioeconomic status, family support, peer support). Results of the Latent Profile Analysis suggest the presence of four different profiles: no resources, only peer, only family, and well-equipped. The association of these profiles with demographic variables, adulthood markers, self-perceived COVID-19 impact, present well-being, and future life perception was investigated. Implications for resilience theory as well as for future interventions are discussed.

**Supplementary Information:**

The online version contains supplementary material available at 10.1007/s12144-022-03658-y.

The COVID-19 pandemic and its consequences at the individual (e.g., unemployment, health risk, postponed weddings, etc.), family (i.e., return to the parental home, worry for parents’ health and job loss, etc.), and social (i.e., social distancing, uncertainty, etc.) levels have made the transition to adulthood harder than before. Emerging adults, i.e., people aged 18–29 years (Arnett, 2014), are indeed dealing with the developmental task of moving from youth to adulthood, and its inherent social challenges: the completion of education, entry into the labour market, leaving the parental home, entry into marriage/cohabitation, and entry into parenthood (Billari & Liefbroer, 2010). In the context of the pandemic, these challenges can become harder. Although emerging adults may be at a lesser risk of COVID-19 severe illness and mortality, their concerns about the present and the future have resulted in higher levels of stress, depression, and anxiety among them. Both longitudinal (e.g., O’Connor et al., 2020) and cross-sectional (e.g., Glowacz & Schmits, 2020) studies have concluded that the negative impact of COVID-19 on mental health and well-being is higher among emerging adults when compared to other age groups.

Consequently, it becomes necessary to understand what can help emerging adults cope and adapt effectively in response to this major stressor, or, in other words, to be resilient (Luthar et al., 2000). Resilience is a salient psychological strength that can reduce the detrimental effect of stressors on psychological health (Arslan, 2016). Resilience has also been conceptualized as a critical psychological resource that refers to the ability to “bounce back” from stress quickly, adapt to new situations flexibly, and even psychologically change in a positive way in the face of adversity (Yıldırım et al., 2021). Recent studies have also stressed the importance of the ability to “bounce back” from stress during the current pandemic period (e.g., Donisi et al., 2021; Jacobson et al., 2021; Yıldırım et al., 2021).

The present study, adopting a person-centred approach, aims to identify different resilience profiles among an international sample of emerging adults surveyed during the pandemic and to test how these profiles relate to demographic variables and well-being outcomes.

## Resilience: a multi-system perspective

For many decades the study of resilience has been characterized by a debate between scholars who conceptualized resilience as a personal characteristic and those who conceptualized it as a dynamic process (Luthar et al., 2000). The first group of researchers considered resilience as a personality trait, often named *ego-resiliency* (e.g., McKay et al., 2019; Yıldırım et al., 2021), “reflecting general resourcefulness and sturdiness of character, and flexibility of functioning in response to varying environmental circumstances” (Luthar et al., 2000; p. 456). The second group of researchers instead conceptualized resilience as the outcome of a process in which different individual and interpersonal factors (e.g., cognitive, emotional, social factors) are activated (e.g., Masten et al., 1990).

More recently, this debate has been resolved by authors proposing a multi-system approach to resilience (Masten et al., 2021). From the perspective of Bronfenbrenner’s ecological systems theory (Bronfenbrenner, 1979), individuals’ lives are shaped by the interactions and coactions of many systems in concert. Specifically, Bronfenbrenner’s theory defines complex “layers” of the environment, each having an effect on individual development. The interaction between biological factors as children mature, their immediate family/community environment, and the societal landscape fuels and steers their development.

Consequently, recent conceptualizations of resilience recognize the interplay of different systems in generating resilience (see Masten et al., 2021 for a review). An evident example is the multi-system model of resilience (MSMR) developed by Liu et al. (2017). This model represents resilience as a tiered system sourced from multiple dimensions. The three multi-dimensional systems are: core resilience, internal resilience, and external resilience. The *core resilience* system comprises intra-individual factors, or trait-like characteristics within an individual that inherently facilitate resilience (e.g., ego-resiliency, biological characteristics); the *internal resilience* system highlights inter-individual and inter-personal characteristics developed or acquired over time (e.g., developed knowledge, positive relationships); and finally, the *external resilience* system contextualizes each individual’s unique circumstances within a larger socio-ecological milieu (e.g., socio-economic status, formal and informal institutions available to the individual). According to Liu et al. (2017), these factors located in different systems can be mobilized in the event of potential challenges or exposures to risks and adversities. In the absence of a stressor or challenge, the systems are still able to delineate pathways to resilience in order to maintain wellbeing (Liu et al., 2017).

## The current study

In the current study we aim to apply the MSMR model to identify resilience profiles present among emerging adults during the COVID-19 pandemic. Specifically, we collected data about dimensions of resilience based on the three different systems in the MSMR model: core resilience system (i.e., ego-resiliency, positivity), internal resilience system (i.e., emotional support received from the family of origin, emotional support received from friends and/or romantic partner), and external resilience system (i.e., religiosity, socio-economic status). In particular, the resilience dimensions we included in the current study were based on previous studies, which revealed the protective effect of the tendency to view life and experiences with a positive outlook (i.e., positivity; Shing et al., 2016), the capacity to recover quickly from difficulties (i.e., ego-resiliency; Yıldırım et al., 2021), reliance on religious beliefs and practices (i.e., religiosity; Reis & Menezes, 2017), emotional support received from peers (i.e., peer support; Agarwal et al., 2020) and from the family of origin (i.e., family support; Chamratrithirong et al., 2020), as well as the economic condition of the family of origin (i.e., socio-economic status; Karmalkar & Vaidya, 2018). Although these studies highlighted the importance of such resilience factors, these factors were not explicitly assigned to any of the three systems of the MSMR model. In the current study, we considered ego-resiliency and positivity as factors belonging to the “core resilience system” as they both fit into the definition of “intra-individual factors, or trait-like characteristics within an individual that inherently facilitate resilience” (Liu et al., 2017; p. 113). Ego-resiliency and positivity can indeed be assimilated into the two broad dimensions of the early temperament, according to Rothbart and colleagues (Derryberry & Rothbart, 1997; Rothbart, 1981): self-regulation and reactivity. Furthermore, we considered the emotional support received from peers (friends and partner) and from the family of the origin as factors belonging to the “internal resilience system” because they well represent resources “that can be fostered, developed, or acquired over time from inter-personal sources, such as family, friends, and personal experiences and encounters.” (Liu et al., 2017; p. 114). Finally, we included religiosity as well as family of origin’s socio-economic status in the outermost external layer of the MSMR model: “external resilience systems”. This third layer is indeed defined as the “socio-ecological context [that] includes larger socio-environmental institutions, both informal and formal, such as socioeconomic status, income, or geographical location.” (Liu et al., 2017; p. 115). We believe that emerging adults’ religiosity is an expression of informal and formal institutions (e.g., church) they were exposed to in their context and that the family of origin’s socio-economic status well represents the socio-economic opportunities present in such context and, consequently, the kind and quality of services the individual had access to (Liu et al., 2017).

These six resilience factors (two for each of the three systems of the MSMR model) are here adopted to identify emerging adults’ resilience profile. Once these profiles are identified, we aim to investigate how these profiles are distributed across the groups based on emerging adults’ demographic variables (country, gender, age) and variables indicating where they are in their transition to adulthood (i.e., adulthood markers: educational status, living arrangement, occupational status, relational status, and parenthood status). Lastly, we investigate the differences in perceived impact of COVID-19, well-being, and future perceptions among the individuals assigned to different resilience profiles.

The MSMR model has been used previously to investigate the resilience of youth during this pandemic; Chavez (2021) applied the MSMR to investigate the stressors that Latinx college students experienced and the factors that contributed to their resilience. Results indicated that religion and familism values were the most important resilience dimensions, fostering feelings of purpose, persistence, and hope. Our study differs from the Chavez (2021)’s study in two important ways: (1) it adopts a person-centered approach and (2) relies on an international emerging adult sample.

Regarding our first point, adopting a *person-centred* approach to analyse the six different resilience dimensions challenges “the assumption that all individuals are drawn from a single population and considers the possibility that the sample might include multiple subpopulations characterized by different sets of parameters” (Morin et al., 2018, p. 805). This results in a classification system that groups emerging adults into distinct profiles or patterns of resilience depending on the available resources. In particular, the person-centred approach allows us to explore different combinations of resilience dimensions that are detectable in an emerging adult sample. Few studies have adopted the person-centred approach to the study of resilience and the majority of those have adopted it to identify profiles of resilience *outcomes* such as well-being and depression (e.g., Cohen et al., 2021). Few studies have applied the person-centred approach to different *dimensions* as potential determinants of resilience. Moran et al. (2017) is one of the few studies using latent profile analysis to examine whether profiles of temperament, accounting for multiple characteristics simultaneously (fear reactivity, frustration reactivity, executive control, delay ability), were related to children’s responses to adversity. Results indicated that profiles with high frustration, low fear, and low delay ability confer a particular lack of resilience for children in high-risk contexts. To the best of our knowledge, no studies have adopted the person-centred approach to identify resilience profiles based on the MSMR model.

Second, our study relied on a cross-national sample; data were collected from six countries (i.e., China, Italy, Lithuania, Portugal, Slovenia, and the US) characterized by diverse pandemic contexts (i.e., severity and spread of COVID-19) at the time of data collection (see Table S1 in the Online Supplementary Materials, OSM, for more details). The international sample allows to take into consideration different pandemic experiences among the sample of emerging adults and to obtain resilience profiles which are not anchored to a specific national context, ensuring greater external validity of the profiles obtained.

## Method

### Procedure

This study is part of a broader research initiative, COVIN (COVid INternational), carried out by Margherita Lanz and colleagues (Lanz et al., 2021). Data were collected from six countries (i.e., China, Italy, Lithuania, Portugal, Slovenia, and the US) between July and September 2020, when the strict protective measures (e.g., lockdown) in response to the first wave of COVID-19 infections were eased in most of these countries. Each research team secured approval of the Institutional Review Board at their home institution before data collection began. To ensure a diverse sample, we used a variety of recruitment techniques, such as university and student mailing lists, posts on social media and relevant social media groups, researchers own participant pools, and snowball recruiting initiated through colleagues, students, and acquaintances who were asked to forward the link to the survey to emerging adults they know. A small percentage of the participants were recruited using Amazon Mturk in the US (0.9% of the entire sample). After providing online consent, participants completed a 15-minute online survey.

### Participants

A total of 2,282 emerging adults signed the informed consent, but some of them (n = 514) completed only the demographic questions and were excluded from this and the previous study published within the same international project (Lanz et al., 2021). In particular, in the current study we adopted only the 1,766 emerging adults who reported information for at least one of the six resilience factors. This inclusion criterion is based on the estimation method we used to manage missing data: full information maximum likelihood (FIML). Including cases with incomplete data as well increases the precision and accuracy of parameter estimates (Enders & Bandalos, 2001).

Included emerging adults were mainly female (77.3%), aged 18–30 years old (M = 23.46, SD = 3.48) and living in China (n = 223), Italy (n = 387), Lithuania (n = 305), Portugal (n = 274), Slovenia (n = 291), and the US (n = 286). Most of the participants were students (64.1%) and half (48.3%) of the total had a full-time or part-time job. Furthermore, 40.0% were single, while the other had a cohabiting (27.2%) or non-cohabiting (32.8%) partner. Only 5.6% of the participants had a child or more.

### Measures

The survey included a series of demographic variables (e.g., country, gender, age), questions about adulthood markers (educational status, pre- and during-COVID living arrangement, occupational status, relational status, and parenthood status) and several aspects of daily life (the full list of measures is available upon request from the first author). We report here only the measures used in the current study.

*Ego-resiliency*. We assessed the trait of resilience (or ego-resiliency) using the Brief Resilience Scale (Smith et al., 2008); it consists of six items (e.g., *I tend to bounce back quickly after hard times*) evaluated on a 5-point Likert scale (1 = strongly disagree; 5 = strongly agree). The total score obtained from this scale is highly reliable (α = 0.87).

*Positivity*. Emerging adults’ tendency to view life and experiences with a positive outlook was assessed through the Positivity Scale (Caprara et al., 2012). This questionnaire is composed of eight items (e.g., *I have great faith in the future*) evaluated on a 5-point Likert scale (1 = strongly disagree; 5 = strongly agree) and generates a highly reliable total score (α = 0.85).

*Religiosity*. To assess the participants’ religiosity, we asked emerging adults to respond to this ad hoc item: “Please indicate how important religion/spirituality is in your daily life using the following scale: 1 = Not at all important; 2 = Slightly important; 3 = Moderately important; 4 = Very important; 5 = Extremely important”.

*Socio-Economic Status*. The socio-economic status of the emerging adults’ family of origin was assessed using a graphic item proposed by Cantril (1965). It consists of a ladder with 10 rungs. Instructions describe the ladder as representing where families stand in the country where the emerging adults grew up. At the top of the ladder are families who are the best off (e.g., those who have the most money, the most education, and the most respected jobs), while at the bottom are families who are the worst off. Respondents clicked on a rung to indicate where they thought their family stood when they were growing up.

*Family and Peer Emotional Support*. We assessed emotional support that emerging adults received from their family of origin and peers (defined as friend and/or romantic partner if they had one) using the Multidimensional Scale of Perceived Social Support by Zimet et al. (1988). In particular, we adopted eight items of the scale: four items to assess the emotional support received from the family of origin (e.g., *I can talk about my problems with my family of origin*) and four items to assess the emotional support received from peers (e.g., *I can count on my friends/my partner when things go wrong*). All items were rated on a 5-point Likert scale (1 = strongly disagree; 5 = strongly agree) and loaded on two highly reliable factors (α = 0.91 and 0.94 for family and peer support, respectively).

*Perception of COVID-19 Negative Impact*. We used a set of six items developed by Conway et al. (2020) to assess the perceived negative impact of COVID-19 pandemic on three dimensions of participants’ lives. Items form three 2-item scales: COVID-19 financial impact (e.g., *I have lost job-related income due to the coronavirus*), measuring the negative impact on financial condition; COVID-19 resource impact (e.g., *It has been difficult for me to get the things I need due to the coronavirus*), measuring the reduced accessibility to needed resources; and COVID-19 psychological impact (e.g., *I have become depressed because of the coronavirus*), measuring the negative psychological consequences of the pandemic. The items were rated on a 5-point scale (1 = completely not true; 5 = completely true), where higher scores indicate a more negative impact of COVID-19 in one’s life. Because the sub-scales are composed of only two items, we evaluated reliability by calculating the Spearman-Brown correlation between the two items in each sub-scale. Inter-item correlations were 0.64, 0.66, and 0.80 respectively for the three COVID-19 impact dimensions (financial, resources, psychological).

*Present Well-being*. To assess participants’ present well-being, we used the Brief Inventory of Thriving (BIT; Su et al., 2014; Sorgente et al., 2020), a mono-dimensional scale assessing how much an individual is thriving (i.e., is healthy, vigorous and successful; Brown et al., 2017). The scale is composed of 10 items (e.g., *My life is going well*) rated on a 5-point Likert scale (1 = strongly disagree; 5 = strongly agree). The total score is highly reliable (α = 0.91).

*Future Life Perception.* To assess the general perception that emerging adults have regarding their future life, we used the Dark Future Scale (Zaleski et al., 2019), which consists of five Likert-type items (1 = completely not true, 5 = completely true). The items measure a tendency to think about future negative expectations and anticipated failures (e.g., *I am afraid that in the future my life will change for the worse*). In this study, we recoded the items to reflect positive expectations of one’s future life in general (i.e., higher scale-scores indicate a more positive perception of the future). The obtained “future life perception” total score was highly reliable (α = 0.87).

### Data analyses

The analyses performed to address our research question consisted of three steps (measurement invariance, latent profile analysis, association between profiles and other variables). The first two steps were performed in Mplus (version 7), and the last step was performed using SPSS (version 20).

**Measurement invariance of the adopted scales.** Before adopting the different scales in a cross-national sample, we confirmed the factorial structure of each scale^1^ and verified whether each structure was invariant across countries. For each scale, we first performed a Confirmatory Factor Analysis (CFA) on the entire sample. The goodness of the model fit was evaluated using the following fit indices: the Comparative Fit Index (CFI), Tucker–Lewis index (TLI), Mean Square Error of Approximation (RMSEA), and Standardized Root Mean Square Residual (SRMR). CFIs and TLIs equal to or higher than 0.90 and RMSEAs and SRMRs equal to or lower than 0.08 indicated acceptable fit. CFIs and TLIs equal to or higher than 0.95, and RMSEAs and SRMRs equal to or lower than 0.05 indicated a good fit (Little, 2013). However, as suggested by Fan and Sivo (2007), these interpretation guidelines related to goodness-of-fit indexes were not treated as “golden rules” or used for inferential purposes, but only as guidelines for descriptive model evaluation, in tandem with parameter estimates, statistical conformity, and theoretical adequacy.

Once a fitting factorial model was found, we verified that this model was equivalent across the six countries under investigation. Measurement equivalence or invariance refers to the consistency in measurement parameters (i.e., factor loading, intercepts, and residuals) across groups and provides a basis for comparing factor means across groups. When the comparison is done across a few groups/nations, the *exact* measurement invariance is usually tested; however, this approach may be problematic in cross-national studies involving many different countries (Byrne & van de Vijver, 2017). In these cases, the *approximate* measurement invariance using the maximum likelihood alignment method (Asparouhov & Muthén, 2014) is recommended. Results of this analysis consist of the parameters (i.e., factor loadings and intercepts) that are non-invariant across the groups under investigation (i.e., countries). If the non-invariant parameters are within the 25% latent mean estimates derived from alignment, the results are considered trustworthy (Asparouhov & Muthén, 2014) and consequently it is possible to assume that the construct has the same meaning across countries as well as to compare factor means across countries.

**Latent Profile Analysis.** To identify the groups (i.e., profiles) that best describe the heterogeneity within the current sample with respect to the different resilience dimensions (ego-resiliency, positivity, religiosity, SES, family support, peer support), we performed a latent profile analysis (LPA), including these resilience dimensions as observed indicators. We examined fit indices of measurement models, beginning with one class and adding classes incrementally. As suggested by Sorgente et al. (2019), selecting the optimal fitting model(s) was based on statistical tests and descriptive measures of relative model fit. We adopted the adjusted Lo-Mendell-Rubin likelihood ratio test (adjusted LMR-LRT; Lo et al., 2001), comparing two consecutive models; if it is not significant, the *k*-profile model is as good as the (*k*-1)-profile model; therefore, the (*k*-1) profile model is preferred according to the parsimony criterion. As descriptive measures of relative model fit, we adopted five information criteria: Akaike information criterion (AIC), Consistent AIC (CAIC), Bayesian Information Criterion (BIC), sample-size adjusted BIC (ssBIC), and Approximate Weight of Evidence criterion (AWE), where lower values indicate better fit.

Once the best model is selected, the quality of its classification (i.e., assignment of people to profiles) had to be evaluated (Masyn, 2013). The most common diagnostic classification is entropy (E_k_), where values closer to 1 indicate a better classification of cases. Furthermore, the quality of the classification is evaluated by checking the class proportion (CP_k_ or π_k_), the modal class assignment proportion (mcaP_k_), average posterior probability (avePP_k_), and odds of correct classification (OCC_k_). Particularly, classification can be considered good when the mcaP_k_ for each profile is included in the 95% CI of the π_k_, avePP_k_ values are equal to 0.70 or higher, and OCC_k_ values are above 5 (Masyn, 2013; Sorgente et al., 2019).

**Relationship between resilience profiles and other variables.** After identifying the best LPA model, we saved the most likely class membership for each individual to have an observed variable representing each participant’s membership in a specific resilience profile. We used this class membership variable to test the relationship between resilience profiles and demographic variables (country, gender, age), variables describing the attainment of adult social roles (educational status, living arrangement, occupational status, relational status, parenthood status), as well as outcome variables (perception of COVID-19 negative impact, present well-being, future life perception).

In particular, a series of chi-square tests were performed in SPSS to describe the resilience profiles in relation to the following variables: country (US vs. Italy vs. Lithuania vs. Portugal vs. Slovenia vs. China), gender (male vs. female), age (18–24 vs. 25–30), education status (still studying vs. completed education), living arrangement before the pandemic (with vs. without parents/grandparents) and during the pandemic (with vs. without parents/grandparents), occupational status (full/part-time employed vs. occasionally employed vs. unemployed), relational status (single vs. in relationship but not cohabiting vs. cohabitation or marriage), and parenthood status (no children vs. at least one child). Finally, we performed one multivariate analysis of variance (MANOVA) and two one-way analysis of variance (ANOVA) to verify if the resilience profile membership affects an individual’s perception of the negative impact of COVID-19 in three life domains (finance, resources, mental health), present well-being, and future life perception respectively. Post-hoc analyses were performed using the Fisher’s LSD (Least Significant Difference) test.

## Results

### Measurement invariance of the adopted scales

Adopted scales had acceptable fit indices when testing their expected model, except for the Brief Resilience Scale, the Positivity Scale, and the Multidimensional Scale of Perceived Social Support, which required the addition of a correlation between two residuals (see Table 1). After adding these correlations, the invariance of each scale’s measurement model was tested using the approximate measurement invariance procedure proposed by Asparouhov and Muthén (2014).

**Table 1:** Multi-group measurement invariance

*Scale*	*Model*	*χ* ^*2*^	*df*	*p*	*RMSEA [90% CI]*	*CFI*	*TLI*	*SRMR*
Resilience	Total sample	259.808	9	< 0.001	0.136 [0.122 0.150]	0.898	0.830	0.047
	- including residual correlation between item 1 and item 2	92.743	8	< 0.001	0.084 [0.069 0.099]	0.965	0.935	0.035
Positivity	Total sample	519.771	20	< 0.001	0.125 [0.116 0.134]	0.877	0.827	0.052
	- including residual correlation between item 1 and item 4	243.382	19	< 0.001	0.086 [0.076 0.096]	0.945	0.918	0.042
Support	Total sample	409.998	19	< 0.001	0.113 [0.104 0.123]	0.931	0.898	0.035
	- including residual correlation between item 7 and item 8	115.308	18	< 0.001	0.058 [0.048 0.068]	0.983	0.973	0.023
Brief Inventory of Thriving	Total sample	415.501	35	< 0.001	0.085 [0.077 0.092]	0.929	0.909	0.039
Dark Future Scale	Total sample	53.472	5	< 0.001	0.074 [0.057 0.093]	0.984	0.967	0.018

Note. *χ*^*2*^  = chi-square test; *df* = degree of freedom; RMSEA = Mean Square Error of Approximation (RMSEA); CI = Confidence Interval; CFI = Comparative Fit Index, TLI = Tucker–Lewis index; SRMR = Standardized Root Mean Square Residual (SRMR)

As reported in Table 2, the percentage of factor loadings as well as intercepts non-invariant across countries were always lower than 25%. This result confirmed that all the scales were sufficiently equivalent across countries and we could proceed with the following steps of the analysis.

**Table 2:** Approximate measurement invariance

Scale	Fixed to zero*	% non-invariant factor loadings	% non-invariant intercepts
Resilience	China	0 out of 36 (0%)	2 out of 36 (5.5%)
Positivity	Italy	0 out of 48 (0%)	8 out of 48 (16.67%)
Support	Portugal	1 out of 48 (2.08%)	4 out of 48 (8.33%)
Thriving	Slovenia	0 out of 60 (0%)	8 out of 60 (13.33%)
Dark Future	Italy	0 out of 30 (0%)	5 out of 30 (16.67%)

*As the free alignment models were “poorly identified” we adopted the fixed alignment. In particular, we fixed to zero the mean of the country that in the free alignment model had the mean closest to zero

### Identification of latent profiles

The six factor scores measuring ego-resiliency, positivity, religiosity, SES, family support, and peer support were saved from the models with invariant measurement model parameters and used as observed indicators of the LPA. As shown in Table 3, the four-profile solution was the preferred one. Despite AIC, CAIC, BIC and ssBIC being poorly informative as they improved when the number of classes increased, AWE and the adjusted LMR-LRT suggested retaining the four-profile solution. In particular, AWE showed the lowest value for the four-profile solution and the adjusted LMR-LRT indicated that adding a new class (5-profile vs. 4-profile solution) did not make a significant difference per *p* < .0001.

**Table 3:** Relative Model Fit Indices for Six Latent Profile Models

Model	AIC	CAIC	BIC	ssBIC	AWE	Adjusted LMR-LRT
1- profile	25675.95	25850.82	25823.82	25738.04	26106.68	
2- profile	25336.34	25556.54	25522.54	25414.53	25878.74	< 0.0001
3- profile	25201.79	25467.33	25426.33	25296.07	25855.86	< 0.0001
4- profile	25051.21	25362.08	25314.08	25161.59	25816.95	< 0.0001
5- profile	24948.41	25304.61	25249.61	25074.88	25825.82	0.0002
6- profile	24342.08	24743.62	24681.62	24484.65	25331.16	< 0.0001

AIC = Akaike information criterion; CAIC = Consistent AIC; BIC = Bayesian information criterion; ssBIC = sample-size adjusted BIC; AWE = Approximate Weight of Evidence criterion; LMR-LRT = Lo–Mendell–Rubin likelihood ratio test

Consequently, the four-profile solution was investigated through the classification diagnostics. As reported in Table 4, this solution satisfied the classification–diagnostic criteria, indicating that the four identified profiles were well differentiated from each other. The only value slightly lower than the cut-off (5) was the OCC of the fourth profile (4.57). Considering that this value approaches the cut-off and that the other values largely satisfied the classification–diagnostic criteria, we retained the four-class solution for the following step of the analysis.

**Table 4:** Classification Diagnostics for the Four-Class Model

Entropy (E)	Class (N)	CP	mcaP	AvePP	OCC
0.78	class 1 (n = 194)	0.11 (0.09-0.15)	0.12	0.85	43.56
	class 2 (n = 265)	0.17 (0.14-0.21)	0.17	0.89	37.15
	class 3 (n = 97)	0.07 (0.04-0.09)	0.07	0.86	86.21
	class 4 (n = 1210)	0.64 (0.59-0.68)	0.64	0.89	4.57

*Note*. E = Entropy; CP = class proportion; mcaP = modal class assignment proportion; avePP = average posterior probability; OCC = odds of correct classification

**Fig. 1:**
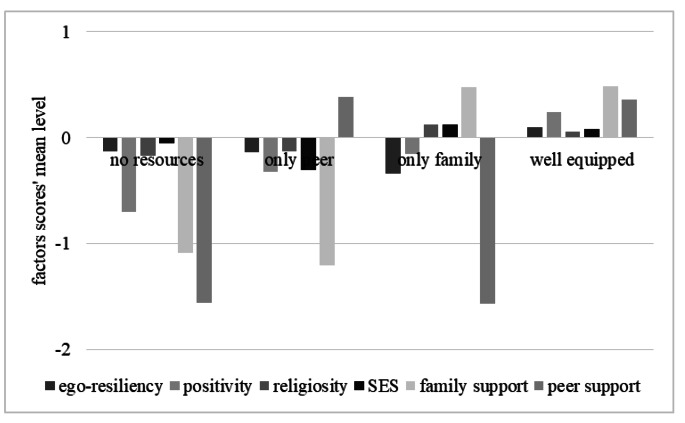
Representation of the four resilience profiles emerging adults showed during the COVID-19 pandemic. Values on the ordinate axis correspond to the factor scores mean level for the six resilience dimensions reported by emerging adults belonging to each profile

The four obtained profiles (see Fig. 1), representing four different patterns in resilience dimensions, were named as follows: no resources (N = 194; 11.0% of the sample), only peer (N = 265; 15.0%), only family (N = 97; 5.5%), and well-equipped (N = 1210; 68.5%).

Emerging adults in the first group (*no resources*) scored lower than the total sample mean (i.e., zero) for all the resilience dimensions we assessed. Members of the second group (*only peer*) scored lower than the total sample mean for all the resilience dimensions, except for the emotional support received from peers. Emerging adults in the third group (*only family*) scored higher than the sample mean only for resources that they have (presumably) derived from their family of origin: their religious faith, the SES of their family, and the emotional support they received from family members. Finally, most of the participants in the fourth group can be described as *well-equipped*, as they affirm possessing all the resilience dimensions under investigation.

### Relationship between resilience profiles and other variables

We saved the most likely class membership for each individual from the four-class solution to use as an observed variable representing each participant’s membership in a specific resilience profile. We further tested how the resilience profiles were related to the demographic variables (country, gender, age), adulthood markers (educational status, pre/during-COVID living arrangement, occupational status, relational status, parenthood status), and outcome variables (perception of COVID-19 negative impact, present well-being, future life perception).

In Table 5 we summarize results from these analyses, while in the OSM we report details for each analysis we ran (i.e., cross-tabulation for chi-square analysis, and mean differences for (M)ANOVA). We found a significant relationship between resilience profiles and country [χ^2^ (15) = 132.032; *p* < .001; Cramer’s *V* = 0.158]; in particular (see Table S2 in OSM), the “no resources” profile was less likely than expected among emerging adults in Portugal and more likely among Chinese emerging adults. The “only peer” profile was less likely than expected among Lithuanian and Slovenian emerging adults and more likely among emerging adults in the US and Italy. The “only family” profile was less likely than expected in China and more likely in Lithuania. Finally, the “well-equipped” profile was less likely than expected in the US, China, and Lithuania and more likely in Portugal and Slovenia.

**Table 5:** Summary of the relationship between resilience profiles and other variables

	No resources	Only peer	Only family	Well-equipped
Country	> China < Portugal	> US, Italy < Lithuania, Slovenia	> Lithuania < China	> Portugal, Slovenia < US, Italy, Lithuania
Gender	> Male < Female	> Female < Male	/	/
Educational status	/	/	> still studying < completed education	/
Living arrangement during the pandemic	/	> without parents < with parents	/	/
Relational status	> single < cohabitation or marriage	> in relationship without cohabiting < single	> single < in relationship without cohabiting	/
Parenthood status	/	/	> at least one child < no children	/
COVID-19 negative financial impact	-	+	/	-
COVID-19 negative resource impact	+	+	-	-
COVID-19 negative psychological impact	+	+	/	-
Present well-being	-	/	/	+
Perception of the future	-	-	/	+

*Note*. Resilience profiles were not significantly related to emerging adults’ age, living condition before the pandemic, and occupational status.

> = more likely than expected; < = less likely than expected; + = profile reporting the highest level of the variable; - = profile reporting the lowest level of the variable; / = no significant trend

We also found a significant relationship between resilience profiles and gender [χ^2^ (3) = 29.802; *p* < .001; Cramer’s *V* = 0.131]. Specifically (see Table S3 in OSM), the “no resources” profile was less likely than expected among female emerging adults and more likely among males; vice versa, the “only peer” profile was less likely among males and more likely among females. In contrast, gender groups were randomly distributed for the other two profiles (only family, well-equipped). Finally, resilience profiles were not significantly related to emerging adults’ age groups [χ^2^ (3) = 1.789; *p* = .617].

Regarding the adulthood markers, resilience profiles were significantly related to emerging adults’ educational status [χ^2^ (3) = 9.450; *p* = .024; Cramer’s V = 0.073] because the “only family” profile was more likely among those who were still in formal education (see Table S4 in OSM). No significant differences were detected for the other three profiles. In addition, resilience profiles were significantly related to the living condition of the emerging adults during the pandemic [χ^2^ (3) = 13.142; *p* = .004; Cramer’s V = 0.090] evidenced by the “only peer” profile being more likely among those who did not live with their parents and/or grandparents (see Table S5 in OSM). No significant differences were detected for the other three profiles.

Finally, the resilience profiles were also significantly associated with relational status [χ^2^ (6) = 46.471; *p* < .001; Cramer’s V = 0.121]) and parenthood status [χ^2^ (3) = 13.062; *p* = .005; Cramer’s *V* = 0.086]. As reported in Table S6 in OSM, the “no resources” profile was more likely among single and less likely than expected among married or cohabitating emerging adults. The “only peers” profile was more likely among emerging adults in a relationship but not cohabiting and less likely among those who were single. In contrast, the “only family” profile was more likely among single emerging adults and less likely among those in a relationship but not cohabiting. Furthermore, the “only family” profile was less likely among those without children and more likely among emerging adults having at least one child (see Table S7 in OSM). We found that resilience profiles were not significantly related to emerging adults’ living conditions before the pandemic [χ^2^ (3) = 2.425; *p* = .489] nor their occupational status [χ^2^ (6) = 8.851; *p* = .182].

Regarding the association of the resilience profiles with outcome variables, we found that emerging adults’ resilience profiles were significantly associated with the perception of the negative impact that the COVID-19 in general [F (9, 3969.576) = 7.331, *p* < .001; Wilk’s Λ = 0.961, partial η^2^ = 0.013], as well as in each of the three life domains: finance [F(3, 1633) = 4.222; *p* = .006; partial η^2^ = 0.008], access to resources [F(3, 1633) = 8.507; *p* < .001; partial η^2^ = 0.015], and mental health [F(3, 1633) = 18.651; *p* < .001; partial η^2^ = 0.033]. In particular (see Table S8 in OSM), the highest level of financial impact was reported by emerging adults belonging to the “only peer” profile; the lowest level of COVID-19 negative financial impact was reported by emerging adults in the “no resources” and “well-equipped” groups and their level was not significantly different from the one reported by “only family” group. In other words, the mean level of financial impact is high for all the participants in our sample, in line with previous publications (e.g., Kämpfen et al., 2020; Swigonski et al., 2021), but this level is even higher for “only peer” emerging adults. The highest levels of resource impact were reported by emerging adults belonging to the “no resources” and “only peer” profiles, while the lowest levels were reported by emerging adults belonging to the “only family” and “well-equipped” profiles. Finally, the highest levels of psychological impact were reported by emerging adults belonging to the “no resources” and “only peer” profiles, while the lowest level was reported by “well-equipped” emerging adults.

Lastly, regarding emerging adults’ present well-being [F(3, 1512) = 87.780; *p* < .001; partial η^2^ = .148] and future life perception [F(3, 1758) = 10.104; *p* < .001; partial η^2^ = .017] we found two significant relationships with the resilience profiles. Specifically (see Table S8 in OSM), the highest level of present well-being was reported by the “well-equipped” emerging adults, while the lowest level was reported by the “no resources” group. The other two groups reported intermediate levels of well-being. The “well-equipped” emerging adults reported the most positive view of the future, while the darkest view of the future was reported by the “no resources” and “only peer” groups.

## Discussion

As people aged 18–29 years were strongly impacted by the pandemic at different levels (individual, family, social; Glowacz & Schmits, 2020; O’Connor et al., 2020), we aimed to identify the resilience profile that is most protective for emerging adults, adopting a multi-system perspective to resilience. Resilience profiles are protective when they buffer the negative impact of COVID-19 and preserve present well-being and a positive view of the future. To thrive in the present (Bachmann et al., 2014) and to have a positive view of the future (Bellare et al., 2019) are two essential ingredients of a positive transition to adulthood. Furthermore, it is also important to identify which demographic characteristics (country, gender, age) and adulthood markers (educational status, pre- and during-COVID living arrangement, occupational status, relational status and parenthood status) are associated with different resilience profiles, in order to more accurately identify vulnerable sub-populations of emerging adults and design tailored interventions accordingly. Toward this end, we performed a Latent Profile Analysis which revealed the presence of four different resilience profiles in a cross-national sample of emerging adults and showed how these profiles were associated with the variables listed above.

Most of the emerging adults (68.5% of the sample) felt that they had sufficient resources to face the adversity posed by the pandemic (at least after the first wave of COVID-19). For each resilience dimension, they indeed reported higher levels than the sample mean. This “well-equipped” group of emerging adults, living mainly in Portugal and Slovenia (two countries with low COVID-19 spread in June 2020; see Table S1), reported the lowest level of negative COVID-19 psychological impact, the highest level of present well-being, and the most positive view of the future.

On the opposite end, 11% of participants reported mean levels of all resilience dimensions below the total sample mean. This “no resources” profile was more likely than expected among Chinese, male, and single emerging adults. This is the most at-risk group as its members reported the lowest level of present well-being and the most negative perception of the future. We can speculate that the vulnerability of this group could also reflect lack of sense of connection beyond the individual, reflected in not having a romantic partner (Pidgeon et al., 2014), as well as cultural context (Chinese; Kim et al., 2008) or gender group (male; Ryan et al., 1998) who are less prone to ask for help. These “no resources” emerging adults may feel they cannot count on others.

Finally, the last two profiles we found are in-between, as they have some resources. On one side, there are emerging adults (15% of the sample) who identified friends and/or romantic partner emotional support as the only resource available. This “only peer” profile was more likely than expected among US and Italian emerging adults, females, and those who had a romantic relationship but did not live with either their partner or family of origin during the pandemic. We can presume these emerging adults as strongly isolated during the pandemic, able to only perceive support of their peers (e.g., their romantic partner or friends). This group also perceived the most negative impact of COVID-19, scoring higher than the “only family” and “well-equipped” groups on all impact dimensions (finance, resources, mental health). While for resources and mental health dimensions, the negative impact perceived from “only peer” participants was equivalent to the one perceived by “no resources” participants, for the financial dimension the “only peer” group had a perception even worse than that of the participants least equipped against stressors. We speculate that this peculiarity of the financial domain may depend on the social comparison generated by the consistent relationship with peers. In particular, literature has stressed that emerging adults evaluate their financial condition in comparison with that of their peers (see the “peer comparison” factor of the Multidimensional Subjective Financial Well-being Scale in Sorgente & Lanz, 2019). Having more contacts with peers than the “no resources” group, emerging adults belonging to the “only peer” group may be more aware of the financial condition of their peers and, consequently, suffer more from this comparison. Future studies should evaluate the plausibility of such speculation.

Despite an average level of present well-being, individuals of the “only peer” group reported (together with the “no resources” group) the darkest perception of the future. This finding suggests that receiving only peer support (likely emotional support provided at distance, due to the pandemic restrictions and the absence of cohabitation with the partner) is not sufficient to face severe adversity such as that of a pandemic. It seems that a single resilience dimension is not sufficient to promote positive outcomes, but rather a variety of resources is needed.

In addition, the importance of receiving support from the older generation is demonstrated by the “only family” group. This group (5.5% of the sample) consists of emerging adults who were more likely Lithuanian, still enrolled in education who were single or had at least one child. We can speculate that emerging adults are more closely tied to the resources received from the family of origin (emotional support, religious faith, socio-economic condition) when they are students, who are single and have not yet fully separated from the family, or are new parents who receive help from the family of origin during a challenging period (e.g., economic support, childcare during the challenging pandemic period, etc.). The effectiveness of this familial pattern of resilience is evident as emerging adults belonging to the “only family” group reported the lowest level of COVID-19 negative resource impact as well as median levels of both present well-being and a positive view of the future. This is in line with literature suggesting that emerging adults who have positive relationships with their parents and family of origin are able to more easily adapt to COVID-19 pandemic-related stresses (Gallegos et al., 2022).

It is worth noting that while family may be effective in preventing negative outcomes, in order to reach the most positive outcomes possible, individual resources are needed too. Indeed, emerging adults who fared best during the pandemic remain those in the “well-equipped” group, i.e., those who could count on both family resources as well as on intra-individual resources (e.g., ego-resiliency, positivity) and the support from peers. This result confirms previous research on the importance of having a variety of resources to overcome adversity (Hobfoll, 1989, 2001). This finding also provides evidence corroborating the multi-system conceptualization of resilience.

Taken together, our findings suggest that (1) the resilience dimensions investigated in this study can help emerging adults face severe adversity, such as the current pandemic; (2) family of origin is a relevant and important source of resilience; (3) emerging adults who fare best are those who also have individual resources, like ego-resiliency and positivity. Consequently, interventions should first focus on identifying and targeting emerging adults who lack resources, including core, internal and external resilience dimensions and second, those whose main source of emotional support comes from peers, as support from emerging adult peers is not sufficient to promote resilience against the pandemic. We can speculate that peers, being emerging adults themselves, are also facing similar struggles with limited external resources during the transition to adulthood and are unable to sustain each other adequately. Furthermore, these interventions need to be systemic, aimed at improving emerging adults’ resources at multiple levels as verified by evidence that the presence of a *variety of resilience dimensions* allow for resilience. Finally, interventions should be provided to emerging adults, regardless of their age, as we verified that these profiles are equally distributed among younger (18–24 years) and older (25–30 years) emerging adults.

Our findings also suggest that emerging adults who fared the worst live in countries which were affected by the virus earlier (China) or more strongly (US and Italy) at the time of data collection. It is likely that today, with the further and prolonged diffusion of COVID-19, the number of vulnerable emerging adults has increased. Furthermore, it seems that emerging adults who are struggling the most are those who are in-between the transition from dependence on the relationship with their family of origin to establishing their own family; they no longer have/perceive support from their family of origin (as, for example, students who are more likely than expected in the “only family support”), neither do they have/perceive the support of a “new family” (as, for example, cohabiting or married emerging adults who are less likely than expected in the “no resources” group).

Findings from the current study should be interpreted in light of its limitations. In particular, the six dimensions of resilience considered (ego-resiliency, positivity trait, religiosity, socio-economic status, family support, peer support) may not be exhaustive, as other studies have identified other dimensions which protect against adversity (e.g., physical exercise, Killgore et al. (2020); emotion-regulation, Barzilay et al. (2020)). Furthermore, the sample is a convenience one and is not fully representative of emerging adults in the six countries studied or emerging adults worldwide. Moreover, the data were collected only at one time point during the pandemic (Summer 2020); this cross-sectional design does not allow us to make causal inferences nor to generalize the results to other waves or time-points over the course of the pandemic. Future studies should thus replicate our study adopting representative and longitudinal data as well as increase the number of resilience dimensions taken into consideration. Finally, as our results indicate that the “no resources” profile is more likely among Chinese emerging adults, future studies should also investigate if this finding could reflect cultural differences in the definition and conceptualization of resilience.

## Conclusion

Because emerging adults (aged 18–29 years) are at a lesser risk of COVID-19 severe illness and mortality but greater risk for impaired mental health and well-being compared to other age groups, the current study aimed to identify resilience profile(s) of emerging adults to detect sub-populations of emerging adults who may be more vulnerable during the pandemic. To weather the challenges of adverse events such as COVID-19, our results suggest that emerging adults, at minimum, need the support from their family of origin and that the most resilient emerging adults are likely to have a much broader base of support, including core, internal, and external resilience dimensions. At the same time, there are many emerging adults (26% in our sample) for whom interventions are needed to see them through, including those lacking resources, as well as those with peer support only.

## Electronic supplementary material

Below is the link to the electronic supplementary material.

Supplementary Material 1

## Footnotes

1 This step was not performed for the *Perception of COVID-19 Impact Scale* as its measurement invariance for the current sample was previously verified in Lanz et al. (2021).

## Statements and declarations

### Access to data and materials

The data and materials that support the findings of this study are available from the corresponding author, AS, upon request.
