# Supplementary material for: Profiles of emerging adults’ resilience facing the negative impact of COVID-19 across six countries
Source: Curr Psychol. 2022 Oct 10:1–13. Online ahead of print. doi: 10.1007/s12144-022-03658-y (PMC9549453; doi:10.1007/s12144-022-03658-y)
Supplement: Supplementary file 1 — Supplementary Material 1 [file 12144_2022_3658_MOESM1_ESM.docx]

**Online Supplementary Materials for**

**Profiles of emerging adults’ resilience facing the negative impact of COVID-19 across six countries**

| Table S1  *COVID-19 epidemiological situation (June 30^th^, 2020) across the six countries in the study* | | | | | | | |
| --- | --- | --- | --- | --- | --- | --- | --- |
| Country | Population (millions) | First registered case | Cases | Deaths | Cumulative incidence | Total cases | Total deaths |
| China | 1,433.78 | December 31st 2019 | 23 | 0 | 0.0 | 84,780 | 4,641 |
| Italy | 60.36 | January 31st | 126 | 6 | 5.2 | 240,436 | 34,744 |
| Lithuania | 2.79 | February 28th | 0 | 0 | 1.5 | 1,816 | 78 |
| Portugal | 10.28 | March 3rd | 266 | 4 | 47.4 | 41,912 | 1,568 |
| Slovenia | 2.08 | March 5th | 4 | 0 | 4.3 | 1,585 | 111 |
| US | 329.06 | January 21st | 41,556 | 336 | 144.8 | 2,590,552 | 126,140 |
| *Notes*. Cumulative incidence = cumulative number of registered COVID-19 cases per 100,000 inhabitants over the past 14 days. Data obtained from the European Centre for Disease Prevention and Control (ECDC, 2020). | | | | | | | |

| Table S2  *Cross-tabulation of resilience profiles and country* | | | | | |
| --- | --- | --- | --- | --- | --- |
|  | Observed values (adjusted residuals) | | | | |
|  | No resources | Only peer | Only family | Well-equipped | Total |
| US | 25 (-1.3) | 70 (**4.9**) | 11 (-1.3) | 180 (**-2.2**) | 286 |
| Italy | 47 (1.0) | 79 (**3.4**) | 16 (-1.3) | 244 (**-2.6**) | 387 |
| Lithuania | 40 (1.3) | 32 (**-2.4**) | 46 (**8.1**) | 187 (**-3.0**) | 305 |
| Portugal | 1 (**-2.3**) | 31 (-1.9) | 8 (-2.0) | 216 (**4.0**) | 274 |
| Slovenia | 24 (-1.6) | 29 (**-2.6**) | 11 (-1.4) | 227 (**3.8**) | 291 |
| China | 38 (**3.1**) | 24 (-1.9) | 5 (**-2.3**) | 156 (0.5) | 223 |
| Total | 194 | 265 | 97 | 1210 | 1766 |
| *Note*. Adjusted residuals in **bold** are those that exceed +/- 2 as suggested by Sharpe (2015). | | | | | |

| Table S3  *Cross-tabulation of resilience profiles and gender* | | | | | |
| --- | --- | --- | --- | --- | --- |
|  | Observed values (adjusted residuals) | | | | |
|  | No resources | Only peer | Only family | Well-equipped | Total |
| Male | 69 (**5.1**) | 41 (**-2.4**) | 24 (0.8) | 244 (-1.9) | 378 |
| Female | 123 (**-5.1**) | 217 (**2.4**) | 73 (-0.8) | 953 (1.9) | 1366 |
| Total | 192 | 258 | 97 | 1197 | 1744 |
| *Note*. We did not include 17 participants who reported “gender queer”. Adjusted residuals in bold are those that exceed +/- 2 as suggested by Sharpe (2015). | | | | | |

| Table S4  *Cross-tabulation of resilience profiles and educational status* | | | | | |
| --- | --- | --- | --- | --- | --- |
|  | Observed values (adjusted residuals) | | | | |
|  | No resources | Only peer | Only family | Well-equipped | Total |
| Still studying | 125 (0.2) | 156 (-1.9) | 74 (**2.6**) | 776 (0.1) | 1131 |
| Completed education | 68 (-0.2) | 109 (1.9) | 23 (**-2.6**) | 434 (-0.1) | 634 |
| Total | 193 | 265 | 97 | 1210 | 1765 |
| *Note*. Adjusted residuals in **bold** are those that exceed +/- 2 as suggested by Sharpe (2015). | | | | | |

| Table S5  *Cross-tabulation of resilience profiles and living arrangement during the pandemic* | | | | | |
| --- | --- | --- | --- | --- | --- |
|  | Observed values (adjusted residuals) | | | | |
|  | No resources | Only peer | Only family | Well-equipped | Total |
| With parents | 127 (0.2) | 139 (**-3.5**) | 64 (1.3) | 780 (1.9) | 1110 |
| Without parents | 58 (-0.2) | 99 (**3.5**) | 22 (-1.3) | 340 (-1.9) | 519 |
| Total | 185 | 238 | 86 | 1120 | 1629 |
| *Note*. Adjusted residuals in **bold** are those that exceed +/- 2 as suggested by Sharpe (2015). | | | | | |

| Table S6  *Cross-tabulation of resilience profiles and relational status* | | | | | |
| --- | --- | --- | --- | --- | --- |
|  | Observed values (adjusted residuals) | | | | |
|  | No resources | Only peer | Only family | Well-equipped | Total |
| Single | 94 (**4.7**) | 75 (**-3.3**) | 44 (**2.2**) | 421 (-1.6) | 634 |
| In relationship, but not cohabiting | 43 (-1.9) | 94 (**2.0**) | 11 (**-4.1**) | 371 (1.7) | 519 |
| Cohabitation or marriage | 28 (**-3.1**) | 77 (1.6) | 31 (1.9) | 295 (-01) | 431 |
| Total | 165 | 246 | 86 | 1087 | 1584 |
| *Note*. Adjusted residuals in **bold** are those that exceed +/- 2 as suggested by Sharpe (2015). | | | | | |

| Table S7  *Cross-tabulation of resilience profiles and parenthood status* | | | | | |
| --- | --- | --- | --- | --- | --- |
|  | Observed values (adjusted residuals) | | | | |
|  | No resources | Only peer | Only family | Well-equipped | Total |
| No children | 187 (1.3) | 249 (-0.3) | 84 (**-3.4**) | 1147 (1.1) | 1667 |
| At least one child | 7 (-1.3) | 16 (0.3) | 13 (**3.4**) | 63 (-1.1) | 99 |
| Total | 195 | 265 | 97 | 1210 | 1766 |
| *Note*. Adjusted residuals in **bold** are those that exceed +/- 2 as suggested by Sharpe (2015). | | | | | |

| Table S8  *ANOVA's post hoc comparisons based upon LSD tests* | | | | |
| --- | --- | --- | --- | --- |
|  | Mean (Standard Deviation) of Factor Scores | | | |
|  | No resources | Only peer | Only family | Well-equipped |
| Covid-19 Financial Impact | 0.03 (0.71)^a^ | 0.19 (0.73)^b^ | 0.05 (0.68)^ab^ | 0.03 (0.71)^a^ |
| Covid-19 Resource Impact | 0.22 (0.83)^a^ | 0.27 (0.81)^ab^ | 0.04 (0.80)^ac^ | 0.04 (0.74)^c^ |
| Covid-19 Psychological Impact | 0.26 (0.88)^a^ | 0.30 (0.83)^ab^ | 0.11 (0.83)^ac^ | -0.05 (0.79)^d^ |
| Present Well-being | -0.85 (0.89)^a^ | -0.29 (.95)^b^ | -0.09 (1.03)^b^ | 0.23 (0.85)^c^ |
| Future Life Perception | -0.10 (0.94)^a^ | -0.24 (0.89)^a^ | -0.11 (0.96)^ab^ | 0.08 (0.94)^b^ |
| *Note*. Means with different superscripts are significantly different from each other. | | | | |
